# Effect of Temperature on Heart Rate for *Phaenicia sericata* and *Drosophila melanogaster* with Altered Expression of the TrpA1 Receptors

**DOI:** 10.3390/insects12010038

**Published:** 2021-01-06

**Authors:** Nicole T. Marguerite, Jate Bernard, Douglas A. Harrison, David Harris, Robin L. Cooper

**Affiliations:** 1 Department of Biology, University of Kentucky, Lexington, KY 40506, USA; Nicole.Marguerite@uky.edu (N.T.M.); jatebernard@gmail.com (J.B.); dough@uky.edu (D.A.H.); 2 520 Ruddles Mill Rd, Paris, KY 40361, USA; dharrisdvm@gmail.com

**Keywords:** TRPA, *Drosophila*, heart, temperature

## Abstract

**Simple Summary:**

Thermal receptors detect temperature changes and can alter the activity of the cells. A subtype referred to as TrpA1 responds to increased temperature and increases expression in the heart of mammals when the heart is injured or is reduced in oxygen. It is not known if this is beneficial or detrimental to the heart. Thus, we examined the effect on heart activity of altering heart expression of TrpA1 in larval fruit flies at varied temperatures. Hearts of normal larvae stopped beating at 37 °C but hearts expressing high levels of TrpA1 stopped beating at 30 °C. In contrast, unmodified larvae of a blowfly species that grows at higher temperatures showed increased heart rate with increased temperature to 37 °C. It is not known if blowflies alter their expression of the thermal receptors. Thermal receptors can also be activated by physical stretch. Thus, it is possible an increase in expression in mammalian hearts within a narrow temperature range could be helpful in maintaining heart rate, as activation of TrpA1 receptors may be modulated by the stretching and relaxing of the heart itself. More research is needed in examining the function of TrpA1 receptors in mammalian hearts.

**Abstract:**

The transient receptor potential (TrpA—ankyrin) receptor has been linked to pathological conditions in cardiac function in mammals. To better understand the function of the TrpA1 in regulation of the heart, a *Drosophila melanogaster* model was used to express TrpA1 in heart and body wall muscles. Heartbeat of in intact larvae as well as hearts in situ, devoid of hormonal and neural input, indicate that strong over-expression of TrpA1 in larvae at 30 or 37 °C stopped the heart from beating, but in a diastolic state. Cardiac function recovered upon cooling after short exposure to high temperature. Parental control larvae (UAS-TrpA1) increased heart rate transiently at 30 and 37 °C but slowed at 37 °C within 3 min for in-situ preparations, while in-vivo larvae maintained a constant heart rate. The in-situ preparations maintained an elevated rate at 30 °C. The heartbeat in the TrpA1-expressing strains could not be revived at 37 °C with serotonin. Thus, TrpA1 activation may have allowed enough Ca^2+^ influx to activate K(_Ca_) channels into a form of diastolic stasis. TrpA1 activation in body wall muscle confirmed a depolarization of membrane. In contrast, blowfly *Phaenicia sericata* larvae increased heartbeat at 30 and 37 °C, demonstrating greater cardiac thermotolerance.

## 1. Introduction

In mammals, cellular metabolism increases when temperature increases above the normal thermal set point. Likewise, for heterothermic animals at higher temperatures, but within their normal functional range, metabolism is increased [1]. Above a critical temperature, organisms are then likely to show that some pathological status and cellular metabolism can be compromised, leading to failure in tissues [2]. In some cases, animals enter a dormant state (i.e., estivate) to decrease metabolism, which is assumed to be a survival technique from overheating due to the raised metabolism at high temperatures [3]. Metabolism from behavioral changes may also increase when exposed to cold in mammals as well as for invertebrates to control body temperature.

It is of interest to know how metabolism increases at a cellular level, which then affects the tissue’s function. In the absence of hormones or other circulating factors in blood or hemolymph and without neural innervation, how is it that cells alter their normal function with changes in temperature? To address such topics, investigation into prokaryotes has helped to understand the optimization of complex biochemical reactions for survival and reproduction [4,5,6,7,8]. Prokaryotes have optimum environmental temperatures for growth, which vary depending on the species. This was examined in correlation to the density of chemical reactions and genome size with temperature [9,10,11].

The increased complexity of eukaryotes with cells to tissues and to systems interacting over temperature ranges is involved, as the systems must be coordinated to function in unison. The sensing of external and internal temperature may be monitored by sensory neurons or cells, which then interact with tissues directly, or through secondary interactions [12]. However, it is of interest to know how isolated tissues may function over varied temperatures to correlate with survival of the organism [2]. Tissues most likely sense thermal changes through biochemical reactions within cells and through receptors on the membrane surface, which can modulate biochemical reactions and add additional thermal sensitivity. Thermal receptors, such as the transient receptor protein-ankyrin, TrpA1 receptors, are ion channels (i.e., Ca^2+^ permeable nonselective cation channel) which alter the ionic flux, leading to altered electrical activity of cells as well as a cascade of other downstream actions [13,14,15]. There are a family of TrpA receptors (i.e., Painless, Pyrexia, TrpA1) known in *Drosophila* to be sensitive to temperatures in the range of 25–45 °C [16]. The TrpA1 receptors contribute to internal thermal sensation by expression in neurons and can result in a behavioral response to environmental temperatures [17]. Thus, we chose to use these receptors for our studies herein. Ionic changes induced by TrpA1 receptors may provide a synergistic or inhibitory action on the inherent thermal response of the biochemical processes. It is unlikely that activation of TrpA1 receptors would not have some modification on cellular processes. 

It is known that TrpA receptors are expressed in mammalian pancreatic cells [18], the spleen, and even in reproductive tissues [19]. There is limited research addressing functions of TrpA receptors expressed in various tissues throughout the mammalian body that are not sensory neurons for internal or external monitoring of temperature. In recent studies, TrpA is noted to be expressed in tissues associated with the heart (vascular smooth muscle, endothelial cells, myocytes) [20] and its expression is noted to be altered as a consequence of cardiac injury and ischemia [21,22]. There is a large family of Trp channels, besides the TrpA subtype, and many are expressed in cardiac tissue [23]. These are differentially expressed in pacemaker cells of the sinoatrial node, atrial and ventricular myocardium, as well as fibroblasts in the heart. The Trp family of channels have various functions from serving as stretch activated channels to regulating second messenger cascades [23]. The TrpA1 expression is altered in cardiac pathological conditions and may serve to be beneficial to enhance contractility due to the receptors also being activated by stretch [24,25]. 

It is of interest to know if the TrpA1 receptors can have an influence on the heartbeat. The over- or under-expression of TrpA1 receptors in a myogenic heart, devoid of neurons, allows a direct effect on the heart tissue to be studied. Therefore, *Drosophila melanogaster* (Meigen, 1830; Diptera: Drosophilidae), with the amenable ability to genetically alter expression in the larval heart, were used to examine physiological consequences and possible effects on heartbeat with altered temperature and altered expression of the TrpA1 receptors. For a review on the development of the insect heart and function, see Bodmer et al. [26]. As in earlier studies, when the heart rate decreased, such as with cold temperatures [27] or with optogenetically induced depressed heart rates [28], the excitatory modulator serotonin (5-HT) was used to test if it could counteract decrement in the heartbeat. 5-HT was also used in this study to examine if its action could counteract the effect in the responses induced by TrpA1 overexpression and activation. 

Since it is established that survival of *D. melanogaster* drastically decreases with temperatures ranging higher than 35 °C [29], while larvae of the medicinal blowfly (*Phaenicia sericata*, Meigen, 1826, Diptera: Calliphoridae) thrive well at temperatures as high as 40 °C, the influence of temperature on the heartbeat of larval (*P. sericata*) was also examined for a comparison. It would be expected that the heartbeat of the *P. sericata* larvae would maintain functionality since the larvae grow and thrive at relatively high temperatures; however, it is possible that the larval crawling movements may be sufficient to circulate the hemolymph in the open circulation of the larvae if the heart did not function. The effects of temperature on physiological function of *P. sericata* is also of therapeutic importance as the larvae are used in wound therapy in humans as well as veterinary care, where environmental temperature varies [30,31]. 

## 2. Methods

### 2.1. Fly Lines

The overexpression in the heart and body wall muscles (i.e., mesoderm) of the TrpA1 receptor was performed by crossing homozygous 24B-Gal4 (III) (BDSC stock # 1767) with female virgins of UAS-TrpA1 (BDSC stock # 26263). Two heart-specific strains, Hand4.2-Gal4 (on II) and Tinc-Gal4 (on II) [32], were also used for expression of TrpA1. Progeny carrying one copy each of GAL4 driver and UAS-TrpA1, referred to as 24B>TrpA1, Hand4.2>TrpA1, and Tinc>TrpA1, were used for physiological analyses. UAS-TrpA1 alone were used for control comparisons.

Tissue-specific RNA interference of TrpA1 was performed using two different transgenic lines: y[1] v[1]; P{y[+t7.7] v[+t1.8] = TRiP.HMS05348}attP2 (BDSC stock # 66905; referred to hereafter as UAS-TrpA1-RNAi#1) and y[1] v[1]; P{y[+t7.7] v[+t1.8] = TRiP.JF02461}attP2 (BDSC stock # 36780; referred to hereafter as UAS-TrpA1-RNAi#2). Virgin females from these UAS-RNAi lines were crossed separately with males of 24B-Gal4, Hand4.2-Gal4, and Tinc-Gal4. Resulting heterozygous animals are referred to using the same notation as above, e.g., 24B>TrpA1-RNAi#1. Control animals for genetic background effects included the UAS-RNAi lines alone, as well as a UAS-GFP line y[1] v[1]; P{y[+t7.7] v[+t1.8] = UAS-GFP.VALIUM10}attP2 (BDSC 35786) generated in the same vector and fly lines. w^1118^ alone, as well as w^1118^ combined with heterozygous 24B-Gal4, UAS-TrpA1-RNAi#1, UAS-GFP, and UAS-TrpA1-RNAi#2, were all examined. All *Drosophila* were obtained from the Bloomington Drosophila Stock Center (BDSC) except the two heart-specific lines (Hand4.2-Gal4 and Tinc-Gal4), which were supplied from Dr. Anthony Cammarato. The heart beating was sufficiently regular and without substantial periods of quiescence to allow for counting of beats over 30 s and multiplied by two for obtaining a value for beats per minute (BPM).

Only early third instar *Drosophila* larvae were used (50–70 h) post hatching. All larvae were maintained at room temperature, ~21 °C, in vials partially filled with a cornmeal–agar–dextrose–yeast medium. Larvae of blowflies (*Phaenicia* (*=Lucilia*) *sericata*) were used as third instar for heart rate measures. The blowflies were raised in the laboratory on beef liver and cow blood agar plates. Dr. David Harris, a veterinarian in Paris, KY, USA, provided sterile medicinal blowfly eggs and larvae for these experiments. 

Two different paradigms were used for raising *Drosophila* strains for heart rate measures. One paradigm was to maintain the lines at 21 °C and the other was to raise the lines at 27 °C after pairing males and females of the crosses or background controls. 

### 2.2. Heart Rates

**Figure 1:**
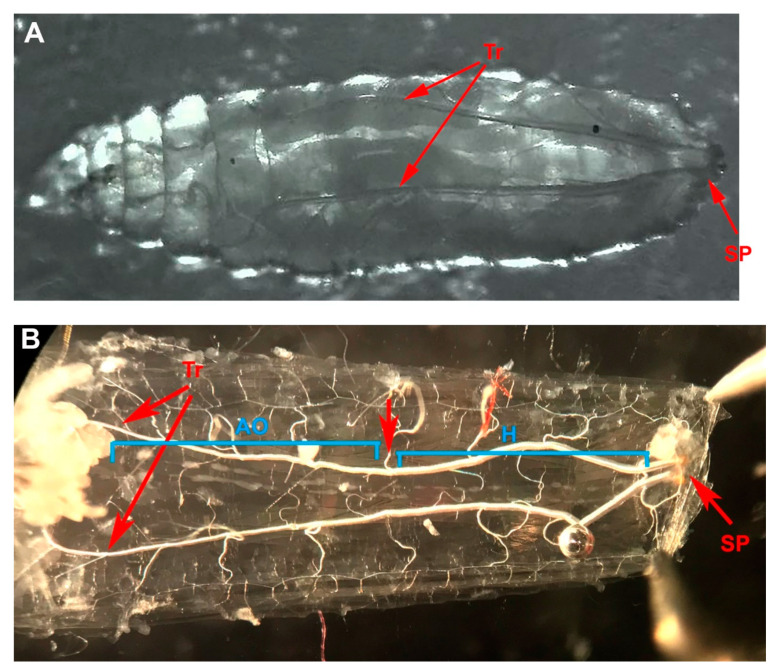
Larval heart tube in a third instar. (**A**) Whole larva is stuck to double-stick tape on a glass slide to observe heart beats with light shining from below. Dorsal view is shown and the heart tube is between the two tracheal tubes. (**B**) The animal is pinned with the dorsal side down. The heart tube is between the two trachea (Tr) running the length of the larvae. The spiracles (SP) are seen on the caudal end. The heart tube has the region of the true heart (H) where the heart generates the electrical rhythm and the aorta (AO) to direct hemolymph toward the head. The preparation is bathed in physiological saline.

**Figure 2:**
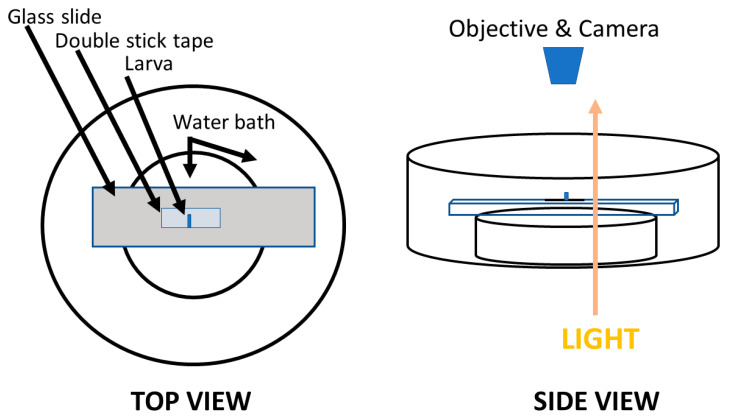
The protocol to observe heart beats in whole larvae. A larva was dried and placed onto double-stick tape with the dorsal aspect facing upward. The slide was placed on a glass Petri dish slightly smaller in diameter than the length of the slide. The smaller Petri dish was placed inside of a wider diameter glass Petri dish to catch spilled over water but also to aid in temperature control. The two dishes were adhered together with surgical wax which held well in a wet environment. The dishes were placed on a base of a microscope with a mirror which can project light through the dishes and onto the larvae.

The heart rates were recorded by video camera and counted by direct visual observation of the recordings. The heart rates obtained for intact larvae were performed by drying the larvae with tissue paper and then placing them on double-stick tape, which was placed on a microscope slide (Figure 1A, also shown as a movie, see Reference [33]). The intact larvae moved a lot and would wiggle at higher temperatures. Bright light was needed to shine through the body as it was easier to see the heart tube and count the heart rate. For this approach, we used light reflected off a mirror and transmitted through the base of the microscope, and the water bath. The light penetrated the slide with the larvae attached to the double-stick tape (Figure 2).

To expose the heart tube to a known medium without the effects of endogenous cardiac modulators present, the larvae were dissected and bathed in saline (Figure 1B). The dissection technique has been previously reported [34]. In brief, the larvae were dissected ventrally and pinned on four corners. The visceral organs were removed, keeping the heart tube intact. The dissection time was roughly 3–6 min. A modified HL3 saline was used (NaCl 70 mM, KCl 5 mM, MgCl_2_.6H_2_O 20 mM, NaHCO_3_ 10 mM, Trehalose 5 mM, sucrose 115 mM, BES (N,N-bis(2-hydroxyethyl)-2-aminoethanesulfonic acid) 25 mM, and CaCl_2_.2H_2_O 1 mM, with pH 7.1 [35]). The chemicals were obtained from Sigma-Aldrich, St. Louis, MO, USA. Dissected larval preparations allow a defined bathing environment free from hormones and peptides in the hemolymph of intact larvae or pupa. Thus, the direct effect of temperature or the addition of 5-HT could be addressed independent of other factors which may fluctuate in the hemolymph, particularly during pupation [35,36,37].

The glass dissection dish was placed on top of a submerged wire platform in water maintained at either 21, 30 or 37 °C. The 30 or 37 °C was controlled by a water bath. The slide was placed on top of a platform, which allowed direct contact of the glass with the water. Each exposure time was first allowed 1 min for heat to transfer through the glass to the bathing saline. Then, the heart was imaged for 2 to 3 min before transferring the slide to the next condition. The whole larvae and dissected larvae were recorded first at 21 °C and then transferred to either 30 or 37 °C. A 1 min incubation time was allowed and then 2 to 3 min of imaging before returning the dish to 21 °C. Upon returning to 21 °C, the dish came in contact with the water bath for 1 min before imaging for the next 2 to 3 min.

### 2.3. Measures of Membrane Potential in Body Wall Muscles

To monitor the transmembrane potentials of the body wall muscle (m6) of third instar larvae, a sharp intracellular electrode (30 to 40 MΩ resistance) filled with 3 M KCl impaled the fiber. An Axoclamp 2B (Molecular Devices, Sunnyvale, CA, USA) amplifier and 1 × LU head stage was used. The bathing saline was initially 21 °C and then exchanged to either 30 or 37 °C while recording the membrane potential. The recordings were made for TrpA1 over-expressing, RNAi expressing, and control animals. The pH was monitored at 30 or 37 °C and a pH of 7.1 was maintained, likely because of the high concentration of BES buffer used in this HL3-modified saline.

### 2.4. Survival with Temperature

Early third instar *Drosophila* were placed in separate dishes with 1 g of the standard cornmeal food. These were raised at 21 °C from eggs. Twenty of each strain were subjected to 24 h at 30 °C and another twenty of each strain were subjected to 24 h at 37 °C. Twenty second instars of *P. sericata* were placed on beef liver in the same incubator at the same time with the *Drosophila* exposed to 30 and 37 °C.

### 2.5. Statistical Analysis

Some data are expressed as raw values. A Sign pairwise test was used to analyze changes in heart rate or membrane potential after changing bath conditions. Because a few datasets are not normally distributed (a number of zeroes in some groups as the heart rate stopped), the non-parametric Sign test was used. When appropriate, paired and unpaired *t*-tests were used. A significant difference is considered at *p* < 0.05. Different symbols were used in the graphs to isolate individual preparations from each other.

## 3. Results

### 3.1. Heart Rate in Whole Larvae with Increased Temperature

**Figure 3:**
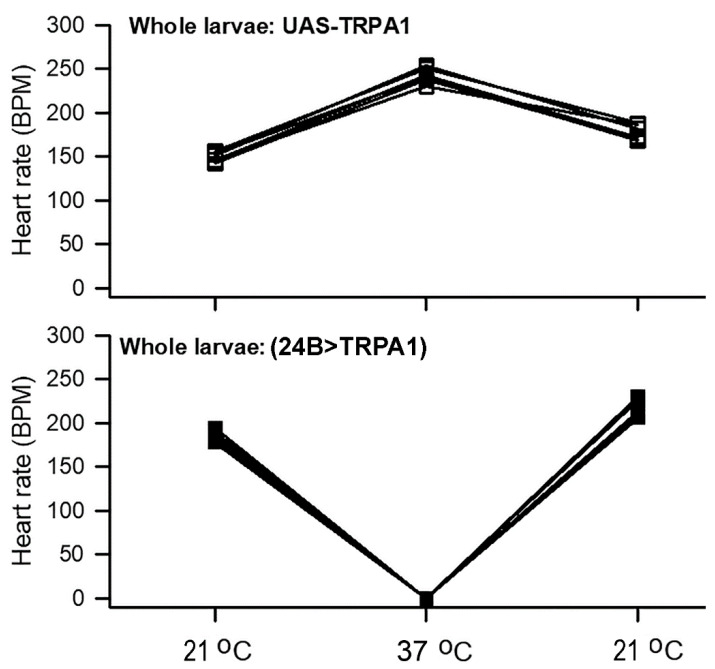
The heart rate for intact larvae. The heart rate increased in UAS TrpA1 and decreased for 24B>TrpA1 from 21 to 37 °C (*p* < 0.05, paired *t*-test). Hearts of 24B>TrpA1 larvae rapidly stopped beating at 37 °C (*p* < 0.05, paired *t*-test). The rates increased upon exposure back to 21 °C.

The heart rate of the intact *Drosophila* larvae from control animals carrying only the UAS-TrpA1 transgene, without a GAL4 driver, increased when animals were shifted from 20 to 37 °C (*p* < 0.05, paired *t*-test, 21 to 37 °C). The heart beats were maintained over the next 3 min at a similar rate. However, the hearts of animals over-expressing TrpA1 in mesoderm (24B>TrpA1) stopped beating at 37 °C (*p* < 0.05, paired *t*-test, 21 to 37 °C) but regained a rhythmic pattern upon returning to 21 °C (Figure 3). Representative movies are shown for whole larva UAS-TrpA1 (Supplementary Movie S1: https://www.youtube.com/watch?v=IFRWd2KmLBA) and for 24B>TrpA1 (Supplementary Movie S2: https://www.youtube.com/watch?v=Wqx_QmzcM0g) undergoing these temperature changes.

### 3.2. Heart Rate in Dissected Larvae with Increased Temperature

**Figure 4:**
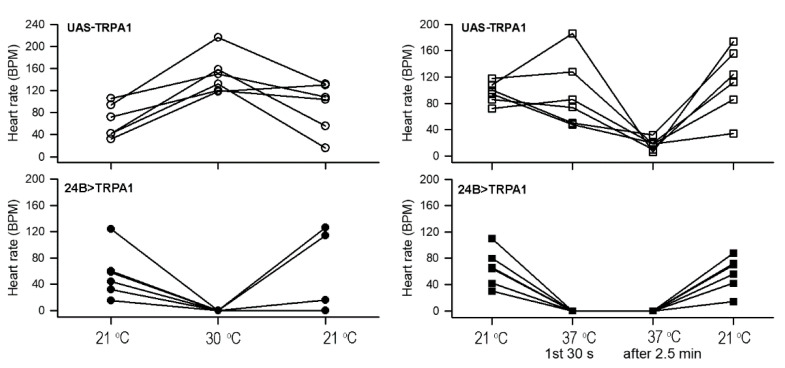
The heart rate for the dissected larvae over expressing TrpA1 in all mesoderm tissue. The UAS-TrpA1 line from 21 to 30 °C (upper left; *p* < 0.05, paired *t*-test). The same strain showed variation upon exposure at 37 °C, with some larvae increasing in rate and others decreasing. After 2.5 min, the rates decreased, but upon exposure to 21 °C, the rates increased (upper right). The 24B>TrpA1 animals’ hearts rapidly stopped beating at 30 °C (lower left) and 37 °C (lower right). The rates would increase upon exposure back to 21 °C. These strains were all raised at 21 °C.

A similar pattern was also observed for the dissected in situ hearts bathed in saline. For these dissected preparations, shifts to 30 or 37 °C were both examined in different larvae. In contrast to the intact larvae, dissected control animals (UAS-TrpA1) displayed a variable initial response upon shift to 37 °C. Some preparations showed increased heart rate while others were reduced (Figure 4). All dissected control animals showed a dramatic reduction in heart rate after two minutes of incubation at 37 °C (*p* < 0.05, paired *t*-test, 37 °C first 30 s to the second recording after 2 min at 37 °C). For all dissected control larvae subjected to a shift to a milder 30 °C incubation, heart rates displayed an increase (Figure 4) (*p* < 0.05, paired *t*-test, 21 to 30 °C). Heart rate was dramatically more sensitive to increased temperatures in the 24B>TrpA1 over-expressing dissected larvae. At 30 °C, the rate rapidly slowed down and within the first 30 s at 37 °C, the beating stopped and stayed depressed over the 3 min incubation at 37 °C. The beating did recover after returning temperature to 21 °C (*p* < 0.05, paired *t*-test, 37 to 21 °C) (Figure 4).

### 3.3. Heart-Specific Over-Expression of TrpA1

**Figure 5:**
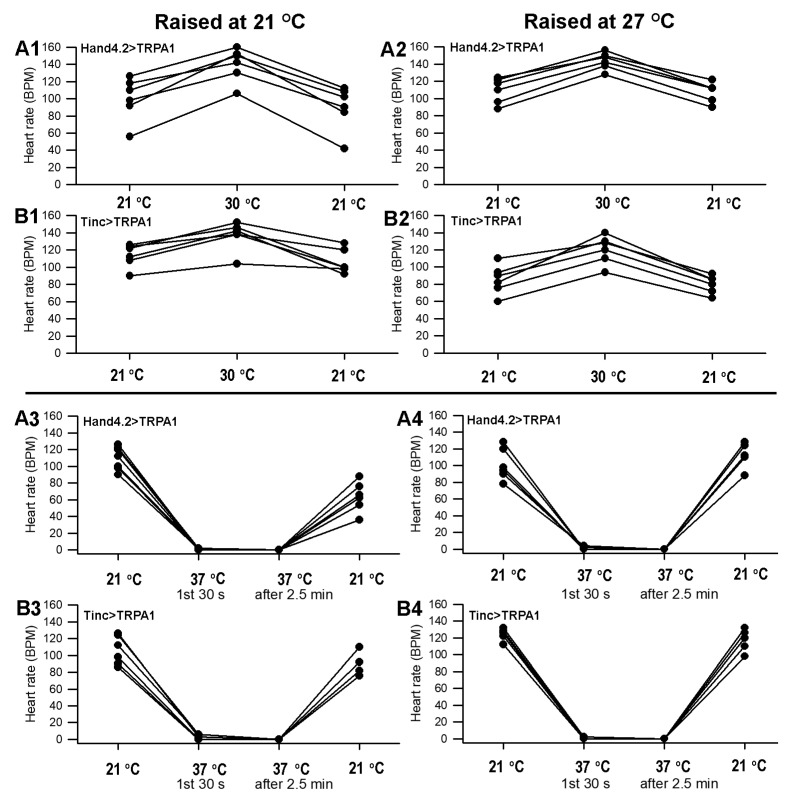
The heart rate for the dissected larvae over-expressing TrpA1 specifically in the heart. The heart rate increased in Hand4.2>TrpA1 (**A1**,**A2**) and Tinc>TrpA1 (**B1**,**B2**) from 21 to 30 °C (*p* < 0.05, paired *t*-test) for both conditions of larvae raised at 21 °C (**A1**,**B1**) as well as at 27 °C (**A2**,**B2**). The strains Hand4.2>TrpA1 (**A3**,**A4**) and Tinc>TrpA1 (**B3**,**B4**) upon exposure at 37 °C all rapidly decreased heart rate and maintained a low rate for 2.5 min. Upon exposure to 21 °C, the rates increased. The same trend was present for the larvae raised at 21 °C (**A3**,**B3**) as well as at 27 °C (**A4**,**B4**). Controls for these strains are illustrated in Figure 4 top panels under the same conditions.

Because 24B-GAL4 drives expression broadly in mesodermal tissue, the effects of TrpA1 misexpression in 24B>TrpA1 larvae could not be specifically ascribed to function in the heart. To determine whether over-expression of TrpA1 specifically in the heart is sufficient for these effects, two heart-specific GAL4 drivers, Hand4.2-GAL4 and Tinc-GAL4, were used to express UAS-TrpA1. Because GAL4-driven expression increases significantly with temperature [38,39,40], these animals and controls were raised at 21 °C as well as 27 °C and examined at temperatures of 21 °C changed to 30 or 37 °C (Figure 5). Similar responses to temperature shifts were observed for both Hand4.2>TrpA1 and Tinc>TrpA1 larvae with heart-specific TRPA1 over-expression at both rearing temperatures. Similar to the responses for general mesodermal over-expression, heart-specific over-expression resulted in rapidly slowed heart rate, and within the first 30 s at 37 °C, the beating stopped and stayed depressed over the next 3 min (Figure 5). The beating did recover after returning temperature to 21 °C (*p* < 0.05, paired *t*-test, 37 to 21 °C). In contrast to responses in larvae with general mesodermal over-expression of TrpA1, heart rates increased for Hand4.2>TrpA1 and Tinc>TrpA1 when shifted to 30 °C, whether raised at 21 or 27 °C (*p* < 0.05, paired *t*-test, 21 to 30 °C).

### 3.4. RNA Interference with TrpA1

Over-expression of TrpA1 in the heart sensitizes larvae to increased temperature but does not address whether endogenous TrpA1 influences this response. To investigate, two different UAS-hairpin RNAi constructs against TrpA1 were expressed broadly in mesoderm and specifically in heart using the same GAL4 drivers used for over-expression. The strains for heart-specific expression in larvae were raised at 27 °C to promote stronger RNAi expression [38,39,40]. The effects of exposing the whole larvae (UAS-TrpA1-RNAi#1 control and 24B>TrpA1-RNAi#1) showed that heart rate increased at 30 °C when increased from 21 °C (Supplementary Figure S1). No differences in the heart rates occurred between the UAS-TrpA1-RNAi#1 and 24>TrpA1-RNAi#1 dissected larva when increasing the temperature from 21 to 30 °C or for the strains for 21 to 37 °C (Supplementary Figure S2). These strains were raised at 21 °C. 

A second RNAi strain (24B>TrpA1-RNAi#2) expressed in mesoderm in addition to 24B>TrpA1-RNAi#1 as well as the backgrounds and controls (UAS-TrpA1-RNAi#1, UAS-TrpA1-RNAi#2, UAS-GFP, and 24B>GFP) were all examined in dissected larvae in temperature changes from 21 to 30 °C and back to 21 °C for larvae raises at both 21 as well as at 27 °C (Supplementary Figure S3). These same lines were also examined in temperature changes from 21 to 37 °C and back to 21 °C for larvae raises at both 21 as well as at 27 °C (Supplementary Figure S4). No significant differences occurred for the control strains under the same conditions. The heart-specific GAL4 drivers (Hand4.2 and Tinc) expressing RNAi also showed no significant differences from the controls to the RNAi expressing lines (Hand>TrpA1-RNA#1, Tinc>TrpA1-RNAi#1, Hand>TrpA1-RNA#2, Tinc>TrpA1-RNAi#2) for larvae examined that were raised at 21 as well as 27 °C (Supplementary Figure S5).

Controls were performed using single transgene-carrying animals and with other transgenes derived from the same vector and genetic background (UAS-GFP). The w^1118^ strain and crosses with w^1118^/+, UAS-TrpA1-RNAi#1/+ did not show any significant differences from UAS-TrpA1-RNAi#1 (Supplementary Figures S2 and S4) when dissected hearts were exposed to 30 °C. Exposing dissected hearts to 37 °C for w^1118^ alone, 24B-Gal4/+, UAS-TrpA1-RNAi#1/+, UAS-GFP/+, and UAS-TrpA1-RNAi#2/+ all showed similar trends with a decrease in heart rates after 2.5 min at 37 °C, with some completely stopping, but none of the strains rapidly stopped upon initial exposure to 37 °C (Supplementary Figure S6) as the TrpA1 overexpression lines did.

### 3.5. Effect of Serotonin on Heart Rate with High Temperature

Since serotonin is well established to increase heart rate (HR) of larval *Drosophila* in cold temperatures (10 °C), as well as room temperature (20–21 °C), we assumed the heart rate would increase with exposure to serotonin once the hearts stopped beating at 37 °C. As soon as the heart beating basically stopped, with only 1 beat or less in 10 s, saline with 10 µM serotonin was flooded over the exposed hearts. The UAS-TrpA1-RNAi#1 larvae showed a burst in rate with serotonin exposure (N = 6; *p* < 0.05, Sign test). However, none of the 24B>TrpA1-RNAi#1 expressing larvae showed any effect by serotonin, not even a single coordinated beat (N = 6; *p* < 0.05, Sign test). There was still the quivering of the heart tube when serotonin was added but no synchronized beating was observed. To demonstrate the effect of serotonin on a heart which just stopped beating while at 37 °C for the 24B-TrpA1-RNAi#1 line, a short movie is provided (Supplementary Movie S3: https://youtu.be/CTdoMDGXNxE ). The few beats before the heartbeat stops exposure to 10 µM serotonin was provided to demonstrate that heartbeat recovers momentarily before terminating again. However, if serotonin is exposed to a heart, which has just stopped for TrpA1 (24B>TrpA1) larvae at 37 °C, the heartbeat does not return (Supplementary Movie S4: https://youtu.be/-zn8tFa3GqE).

**Figure 6:**
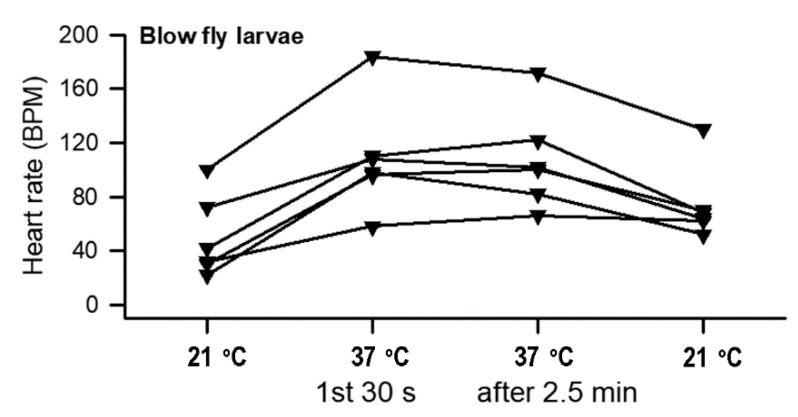
Heart rate in dissected and pinned out *P. sericata* to expose them to saline. The hearts were devoid of hormonal factors in the dissected preparations and demonstrated an increase in rate with high temperatures (*p* < 0.05, paired *t*-test 21 to the initial 37 °C).

Since the saline used was known to maintain the *Drosophila* heartbeat, we assumed the same saline would be sufficient for maintaining the heart rate for *P. sericata* larvae. The hearts did beat well in the same saline. Thus, the effect of higher temperatures on the exposed in situ hearts was able to be examined. The heart rate for *P. sericata* increased upon exposure to saline at 37 °C (*p* < 0.05, paired *t*-test) followed by a decreased rate upon returning the temperature to 21 °C (Figure 6). Thus, the saline developed for *Drosophila* physiology can be used for *P. sericata.*

### 3.6. Resting Membrane Potential of Body Wall Muscles

**Figure 7:**
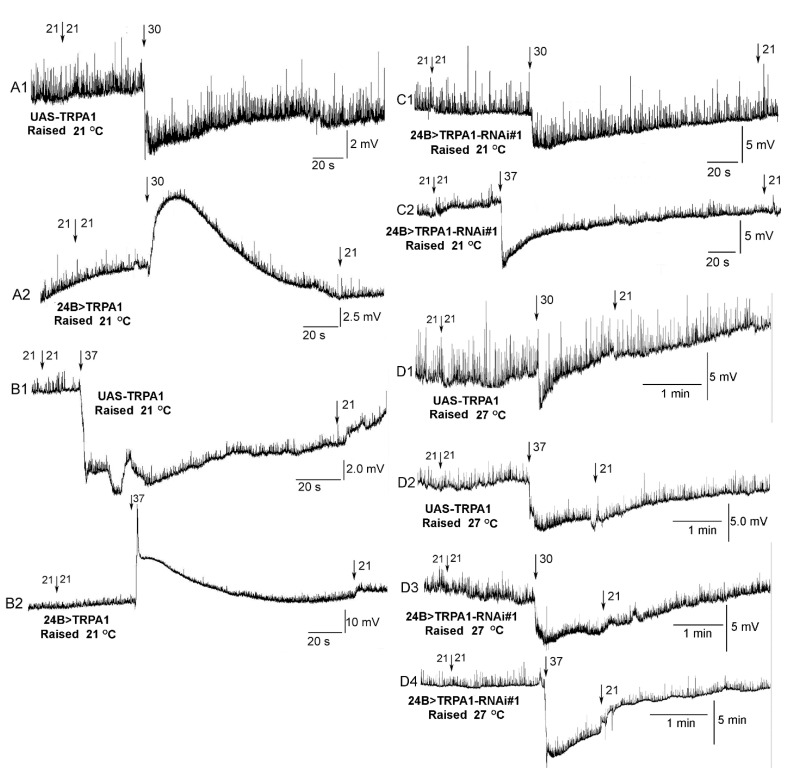
Membrane potential of body wall muscles in TrpA1 overexpressing and RNAi larvae at 30 and 37 °C. The background line of UAS-TrpA1 at 30 °C (**A1**) and 37 °C (**B1**) showed hyperpolarization. The 24B>TrpA1 expressing line at 30 °C (**A2**) and 37 °C (**B2**) showed transient depolarization. In accordance with the background UAS-TrpA1, the UAS-TrpA1-RNAi#1 at 30 °C (**C1**) and 37 °C (**C2**) showed transient hyperpolarization. The lines raised at 21 or 27 °C (**D1**–**D4**) had similar responses. By switching the saline bath from 21 to 21 °C, we controlled for mechanical disturbance of changing the bathing media. Arrows indicate where the saline bath was exchanged. N = 6 for each line for each temperature and the trends were consistent with hyperpolarization (UAS-TRPA as well as UAS-TrpA1-RNAi#1, Sign test, *p* < 0.05) or depolarization (24B>TrpA1, *p* < 0.05, Sign test).

In order to confirm TrpA1 expression and that RNAi was in fact inhibiting expression, the effect of increased temperature was examined on the resting membrane potential of m6 body wall muscle in a *Drosophila* line. Since 24B-Gal4 is targeted for expression in mesoderm tissue, which in *Drosophila* includes the heart as well as body wall muscles, the effect on the body wall muscles with temperature serves as an additional confirmation for gene regulation. The parental background control (UAS-TrpA1) hyperpolarized when exposed to 30 or 37 °C (Figure 7(A1,B1)). The 24B>TrpA1-RNAi#1 also hyperpolarized at both temperatures (Figure 7(C1,C2)) while the 24B>TrpA1 over-expressing lines depolarized for 30 and 37 °C (Figure 7(A2,B2)). The effects were consistent for six of six preparations in each condition (Sign test, *p* < 0.05).

### 3.7. Survival with Temperature

The third instar larvae of the *24B>TrpA1* placed at 30 and 38 °C all died within 24 h (N = 20 for each *p* < 0.05, Sign test). The background (UAS-TRPA) also all died at 38 °C within 24 h (N = 20 for each *p* < 0.05, Sign test). However, only 2 of the 20 larvae of the background (UAS-TRPA) died at 30 °C within 24 h. All the *P. sericata* larvae survived at 38 °C in the beef liver food provided within the 24 h and become notably larger, which indicates a healthy environment for them. In a different laboratory setting, larva raised for medicinal use in veterinary care where there is a high density of larvae (~100′s in a 30 × 30 cm plastic tube), also feeding on beef liver, are known to reach temperatures of 40 °C with continued growth and survival. Thus, the majority of background *Drosophila* strain survived at 30 °C and a 100% survival rate of *P. sericata* occurred at 38 °C (N = 20 for each *p* < 0.05, Sign test).

## 4. Discussion

This study demonstrated that *Drosophila* larvae that over-express the TrpA1 receptor in the heart show an increase in heart rate as the temperature is raised from 21 °C to higher temperatures and will cease beating rapidly at 37 °C. In contrast, blowfly larvae showed increased heart rate at 37 °C that was sustained during the three-minute incubation period. However, the RNAi strains of TrpA1, as well as the background controls, maintained heartbeat at 30 °C with a general reduction at 37 °C after two and half minutes of exposure. It is important to note that the dissected larval preparations are free from influence of components in the hemolymph so the direct effect of activation of the heart by temperature or the addition of 5-HT could be assessed. While the heartbeat was in stasis, serotonin did not rejuvenate the synchronized heartbeat of TrpA1-expressing strains but did rejuvenate the TrpA1-RNAi strains for a short time. The heartbeat regained function with acute exposures of less than 5 min at 37 °C in all lines when the temperature was returned to 21 °C. Expression of TrpA1 in body wall muscle did result in depolarization with exposure to 37 °C saline, whereas the background strains and RNAi strains presented a hyperpolarization with heated saline. This was expected since the E_K_ would likely be at more negative values. In addition, freely moving larvae of background and RNAi lines survived at 30 °C over 24 h, while the over-expressing TrpA1 (24B>TrpA1) larvae died. Intact and dissected larvae of *P. sericata* showed an increase in heart rate at 30 and 37 °C as well as survival and growth of larvae at temperatures of 40 °C.

The response of heart rate to a mildly elevated temperature of 30 °C was significantly different for 24B>TrpA1 larvae, in which over-expression extended throughout the mesodermal, compared with heart-specific over-expression in Tinc>TrpA1 and Hand4.2>TrpA1 larvae, as shown in Figure 4 and Figure 5. TrpA1 over-expression in all mesoderm resulted in a dramatic decrease in heart rate at 30 °C, whereas heart-specific over-expression yielded larvae whose heart rates increased when shifted to 30 °C. These differences may reflect sensitivity of some other mesodermal tissue to TrpA1 over-expression that compromises heart activity. For example, the alary support muscles pull on the heart tube, which may have had an impact on the heart rate. Such effects could be due to activity of the TrpA1 in non-heart mesoderm at the time of the temperature shift or a consequence of a developmental perturbation that has compromised heart activity. Alternatively, the 24B driver may lead to greater accumulation in the heart of the over-expressed TRPA1 than achieved by either of the heart-specific drivers. Accumulation of more active TRPA1 in 24B>TrpA1 may then hypersensitize those larvae to raised temperature, leading to heartbeat cessation at a lower temperature than the Hand4.2>TrpA1 and Tinc>TrpA1 larvae. The data here do not distinguish between the potential spatial, temporal, and quantitative causes of observed differences in these animals.

It is interesting that temperature-sensitive TrpA1 receptors are present in mammalian hearts and other non-neuronal cells, and that in cardiac failure, there is an upregulation in expression [23]. The rise in expression levels possibly leads to a means of adaptive compensation to help increase in contractility, but too much upregulation in expression may block the beneficial effect in a lack of homeostatic regulation in cytosolic Ca^2+^. Since there are other types of TRP receptors expressed in the heart with different regional expression and functions affecting cellular function (see review by Hof et al. [23]), the increased expression of TRPA may aid in redundancy of TRP channels which can be recruited to be expressed when cardiac function is compromised. Slightly increasing [Ca^2+^] background in compromised atrial and ventricular myocytes could not only alter inotropic actions on the heart, but also alter chronotropic actions if the TrpA1 receptors were also increased in the pacemaker cells. Changes in Ca^2+^ influx or background levels can also be a hindrance if the levels are not well-regulated in the larval heart, as well as in mammals [20,41]. Considering the larval heart could not beat normally or even synchronize at a faster rate with overexpression and strong activation of the TrpA1 receptors illustrates the deleterious effect possible on cardiac function. Given that the larvae died with over-expression after 24 h of heat stimulation, as compared to RNAi and background strains, at the same temperature of 30 °C or the TRPA over-expressers maintained at 20–21 °C reveals that the TrpA1 expression and activation can be lethal. 

Interestingly, heart rates monitored in the early pupal (P1) stage of *Drosophila* are similar for 21 °C and higher temperatures [42], but long-term studies for survival are still needed for pupae and adults. The high degree of variability in the heart rate in dissected larval preparations may be due to saline lacking in hormones and peptides which are present in the hemolymph of intact larvae, pupae, and adults; however, the defined saline allows control of the salts and removes stress factors that could be released into the hemolymph helping to maintain low variability in intact organisms [34,37]. 

Exposure of serotonin increases the heart rate in *Drosophila* (Canton S strain) larvae at 10 °C, as well as at 21 °C [36,43,44]. In addition, the RNAi and background lines used in this study increased heart rate with exposure to 10 µM serotonin, but the over-expressors which ceased beating at 30 °C did not respond. Past studies indicate that serotonin activates a phospholipase C (PLC)-inositol 1,4,5-trisphosphate (IP3)-protein kinase C (PKC) pathway through 5-HT2 receptor in *Drosophila* larval heart [43]. The PLC activation can activate two second messengers, inositol tri-phosphate (IP3) and diacylglycerol (DAG), and the rise in DAG can activate protein kinase C (PKC); whereas, IP3 binding to IP3 receptors on endoplasmic reticulum (ER) will cause Ca^2+^ release into cytosol [43]. It is well established that the sarco-endoplasmic reticulum Ca^2+^-ATPase (SERCA) is important to regulate the heart rate [45] and a cytoplasmic Ca^2+^ influx will result in an increase in heart rate [28]. Thus, if the serotonin is increasing cytoplasmic Ca^2+^ from the ER, in addition to Ca^2+^ influx from the TrpA1 activation which has already compromised the heart contraction, then it is reasonable that the addition of serotonin would not increase a synchronized heartbeat. Even slight depolarization of cardiac muscle by activating an anion-conducting channel rhodopsins (GtACR1/2) results in increased heart rate. But strong activation of the light-sensitive channel depolarizes the myocytes further and results in a similar effect with the full contraction ceasing and then causing the heart tube to quiver. This was shown in a video (Supplementary Video S5—of Stanley et al. [44])

The mechanoreceptor of the TRP channel Painless [46] expressed in *Drosophila* larval heart was demonstrated to result in cessation of the heartbeat with activation by heat or by mechanical stimulation [41]. Thus, the TrpA1 and Painless receptors both appear to modulate the larval heart. Since the genome of *Drosophila* appears to contain 16 different TRP channels [47], a variety of TRP channels could be genetically expressed in the heart, as well as with ranges of activation to reach an ideal ability to increase or decrease the heart rate, possibly with mechanical, thermal, or chemical stimulation. It appears that a balance of activating the larval heart with mechanical stimulation is important to maintain the heartbeat over time, as static exposure to the heart will not maintain a consistent beat for hours as compared to a peristaltic stimulation of the heart tube [48]. Interestingly, not all TrpA1 channels in vertebrates are thermal-sensitive, but some are mechanosensitive [49]. Also, not all mammalian TRP channels are sensitive to mechanical deformation of the membrane [50]. However, we did demonstrate the over-expression of TrpA1 depolarized body wall muscles, whereas the background controls and RNAi knockdowns of TrpA1 in the larval *Drosophila* body wall muscle responded similarly to thermal changes.

Altering temperature has an impact on the resting membrane potential of cells. This is evident even without the presence of TrpA1 receptors due in part to the changes on the equilibrium potential of leak channels [51]. Such changes can lead to effects on the activation of voltage-gated ion channels. Thus, altering the temperature can have indirect actions on potential TrpA channels or in combination with the effects of the TrpA1 receptors. The effect of temperature on parental background strain (UAS-TrpA) and the RNAi strains hyperpolarizes the body wall muscles and likely the cardiac myocytes. Hyperpolarization of the membrane potential with increased temperatures are documented in various studies in arthropods. The relative change is approximately 1 to 1.3 mV/1 °C change in body wall muscles of crustaceans [51,52,53,54,55].

This also occurs when the anion pump halorhodopsin (eNpHR) is activated [44]. Thus, one might expect heart rate to slow down in larvae not expressing TrpA1 with increased temperature if the resting membrane potential becomes more hyperpolarized; however, the heartbeat increased in frequency in a synchronized manner. Thus, the actions on biochemical reaction and regulation of ionic fluxes must override the hyperpolarization effects on membrane potential. It is challenging to record the membrane potential or image ion-sensitive indicators in a contracting heart, but this would help to better understand the ionic fluxes [48]. Future studies in the expression profiles of TrpA1 and potential modified forms of TrpA receptors in various insect species, which inhabit different thermal environments, would be of interest. Of the many species of *Drosophila,* some thrive in warmer environments better than others [29,56]; however, at 30 °C, we found only a 40% survival rate of larvae of *D. melanogaster* (UAS-TrpA1 background line) within 24 h, whereas the larvae of blowfly (*P. sericata*) continue to develop at 40 °C. *P. sericata* do show thermal sensitivity, but likely regulate the ionic flux from activating TrpA receptor in neurons and other tissues differently than *D. melanogaster* to allow cells to function properly and insure whole animal survival. Heart rate modulation at increased temperature and body wall muscle membrane potential was similar in control animals and larvae expressing a TrpA1 RNAi transgene in heart or all mesoderm. The lack of an alteration in phenotypes could be because the level of TrpA1 knockdown in these animals was insufficient to compromise function of the gene in these tissues. However, two different TrpA1 RNAi transgenes were tested here, one of which has been used to elicit loss-of-function phenotypes by other researchers [57,58,59,60]. Combined with the 24B-GAL4 that showed strong effects for over-expression, it would be somewhat surprising if the RNAi lines were not achieving significant reduction of endogenous TrpA1. Alternatively, the lack of altered phenotypes in knockdown larvae may be because TrpA1 does not normally play a significant role in the response of heart rate and body wall muscle membrane potential to elevated temperatures. TrpA1 may either not be expressed in these tissues or its function may be compensated by another gene product. Additional studies will be needed to distinguish these possibilities.

## Data Availability

All data generated or analyzed during this study are included in this published article.

## Supplementary Materials

The following are available online at https://www.mdpi.com/2075-4450/12/1/38/s1, Figure S1: The heart rate for intact larvae. The heart rate increased in UAS-TrpA1-RNAi#1 and in 24B>TrpA1-RNAi#1 from 21 to at 37 °C (*p* < 0.05, Paired *t*-test). There is no significant difference from the background to the RNAi strain. Figure S2: The heart rate for the dissected background strain and TrpA1-RNAi expressed in all mesoderm. The heart rates increased in UAS-TrpA1-RNAi#1 from 21 to at 30 °C (*p* < 0.05, Paired *t*-test). The same strain showed variation upon exposure at 37 °C, with some larvae increasing in rate and others decreasing. After 2.5 min, the rates also continued to show variation with some preparations increased rates while other decreased. There is no significant difference from the background to the RNAi larvae. These animals were all raised at 21 °C. Figure S3: The heart rate for the dissected background and expression of TrpA1-RNAi in mesoderm for larvae raised at 21 °C and 27 °C for exposure to 30 °C. The heart rate increased in UAS-TrpA1-RNAi#1 and 24B>TrpA1-RNAi#1 from 21 °C to at 30 °C for larvae raised at 21 °C and 27 °C (*p* < 0.05, Paired *t*-test). The UAS-TrpA1-RNAi#2, 24>TrpA1-RNAi#2, UAS-GFP and 24B>GFP strain showed a lot of variation upon exposure 30 °C for being raised at 21 °C or 27 °C. There is no significant difference from background strains compared to the ones crossed to the 24B strain. Figure S4: The heart rate for the dissected background and expression of TrpA1-RNAi in mesoderm for larvae raised at 21 °C and 27 °C for exposure to 37°C. The heart rate showed a lot of variation upon the initial exposure to 37 °C for most of the strains; however, after two and half minutes the rates generally decreased for all the strains. The UAS-TrpA1-RNAi#2, 24>TrpA1-RNAi#2, UAS-GFP and 24B>GFP strains showed a lot of variation upon exposure 37 °C for being raised at 21 °C or 27 °C. There is no significant difference from background strains compared to the ones crossed to the 24B strain. Figure S5: The heart rate for the dissected strains expressing TRPA-RNAi in heart specific tissue. The heart rate increased in Hand4.2>TrpA1-RNA#1 (A1, A2) and Tinc>TrpA1-RNAi#1 (B1, B2) from 21 °C to at 30 °C (*p* < 0.05, Paired *t*-test) for both conditions of larvae raised at 21 °C (A1,B1) as well as at 27 °C (A2,B2). The stains Hand4.2>TrpA1-RNAi#1 (A3, A4) and Tinc>TrpA1-RNAi#1 (B3, B4) upon initial exposure at 37 °C showed quite a bit of variation. However, at 37 °C after 2 and half minutes all rapidly decreased heart rate. The same trends were present for the larvae raised at 21 °C (A3,B3) as well as at 27 °C (A4,B4). There is no significant difference from background strains compared to the ones crossed to the heart specific strains. Figure S6: Controls for the expression of the RNAi strains crossed with the W118 strain for dissected preparations. The UAS-TrpA1-RNA#1/+ showed similar trends as UAS-TrpA1 in an increase in heart rate at 30 °C. The 24B>TrpA1-RNAi#1 as well as the backgrounds and controls (UAS-TrpA1-RNAi#1, UAS-TrpA1-RNAi#2, UAS-GFP, and 24B>GFP) all showed variation upon initial exposure to 37 °C and showed a decrease after two and half minutes at 37 °C. No significant difference occurred among the strains.
