# Supplementary material for: Effect of Temperature on Heart Rate for Phaenicia sericata and Drosophila melanogaster with Altered Expression of the TrpA1 Receptors
Source: Insects. 2021 Jan 6;12(1):38. doi: 10.3390/insects12010038 (PMC7825143; doi:10.3390/insects12010038)
Supplement: Supplementary file 1 [file insects-12-00038-s001.pdf]

Supplemental Figures

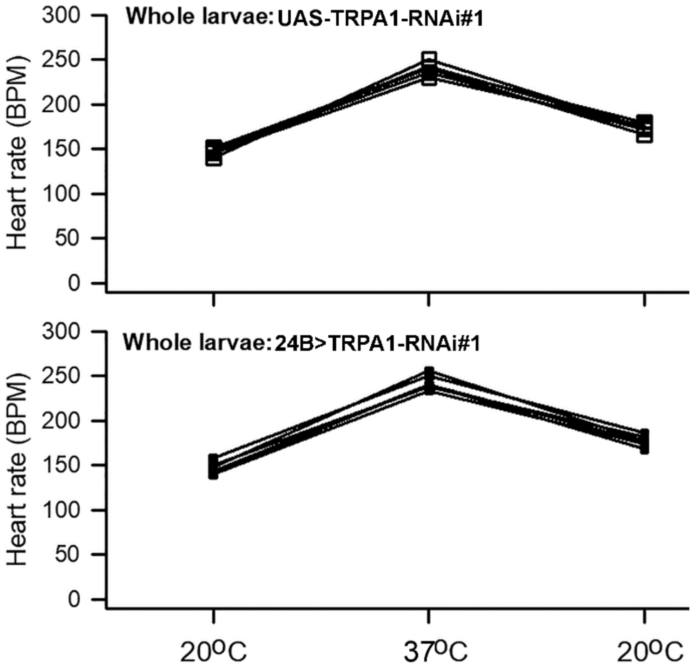

S1: *The heart rate for intact larvae. The heart rate increased in UAS-TrpA1-RNAi#1 and in 24B>TrpA1-RNAi#1 from 21 to at 37°C (P<0.05, Paired T-test). There is no significant difference from the background to the RNAi strain.*

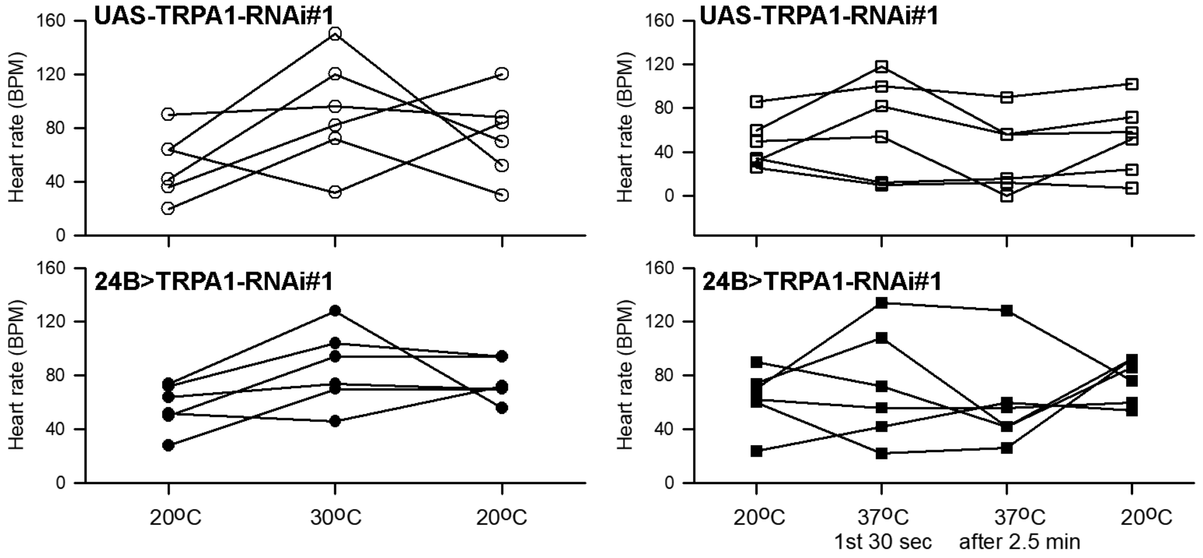

S2: *The heart rate for the dissected background strain and TrpA1-RNAi expressed in all mesoderm. The heart rates increased in UAS-TrpA1-RNAi#1 from 21 to at 30°C (P<0.05, Paired T-test). The same strain showed variation upon exposure at 37°C, with some larvae increasing in rate and others decreasing. After 2.5 minutes, the rates also continued to show variation with some preparations increased rates while other decreased. There is no significant difference from the background to the RNAi larvae. These animals were all raised at 21 °C.*

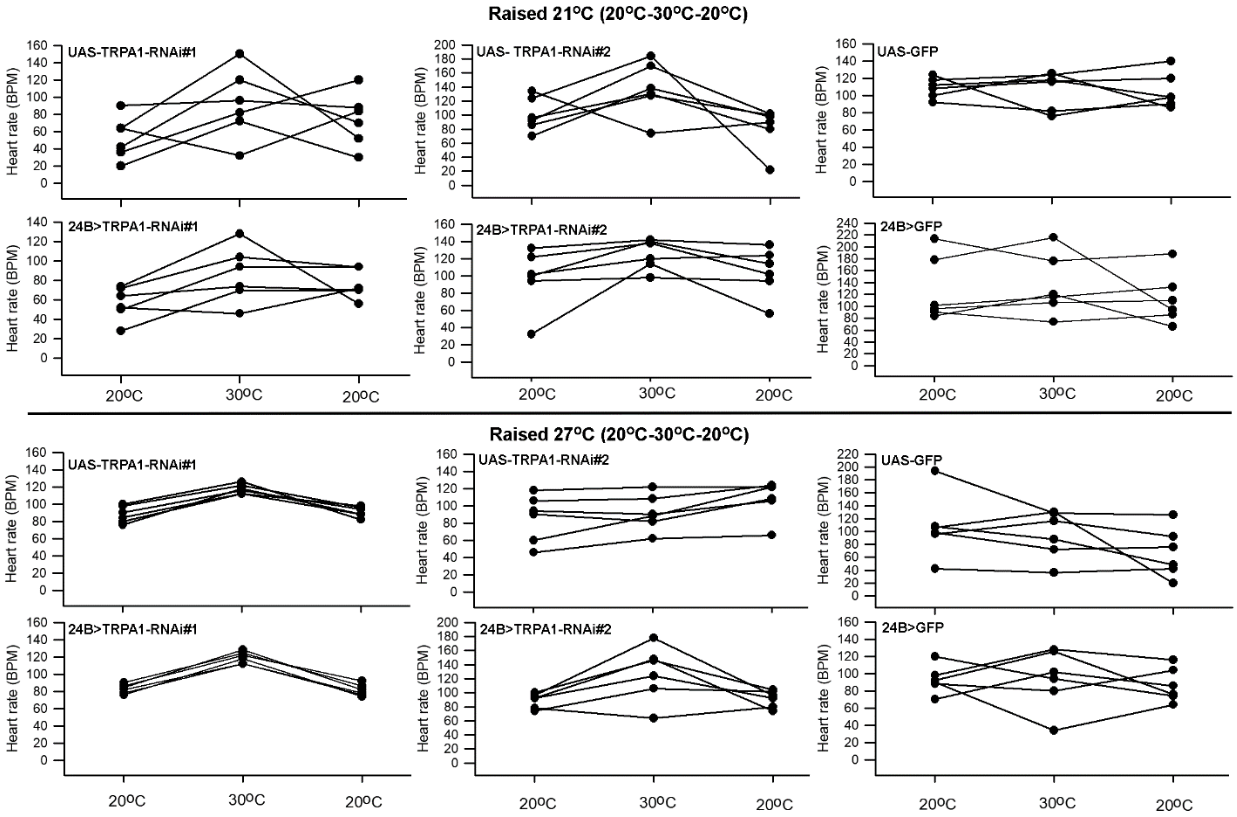

S3: *The heart rate for the dissected background and expression of TrpA1-RNAi in mesoderm for larvae raised at 21°C and 27°C for exposure to 30°C. The heart rate increased in UAS-TrpA1-RNAi#1 and 24B>TrpA1-RNAi#1 from 21°C to at 30°C for larvae raised at 21°C and 27°C (P<0.05, Paired T-test). The UAS-TrpA1-RNAi#2, 24>TrpA1-RNAi#2, UAS-GFP and 24B>GFP strain showed a lot of variation upon exposure 30 ºC for being raised at 21°C or 27°C. There is no significant difference from background strains compared to the ones crossed to the 24B strain.*

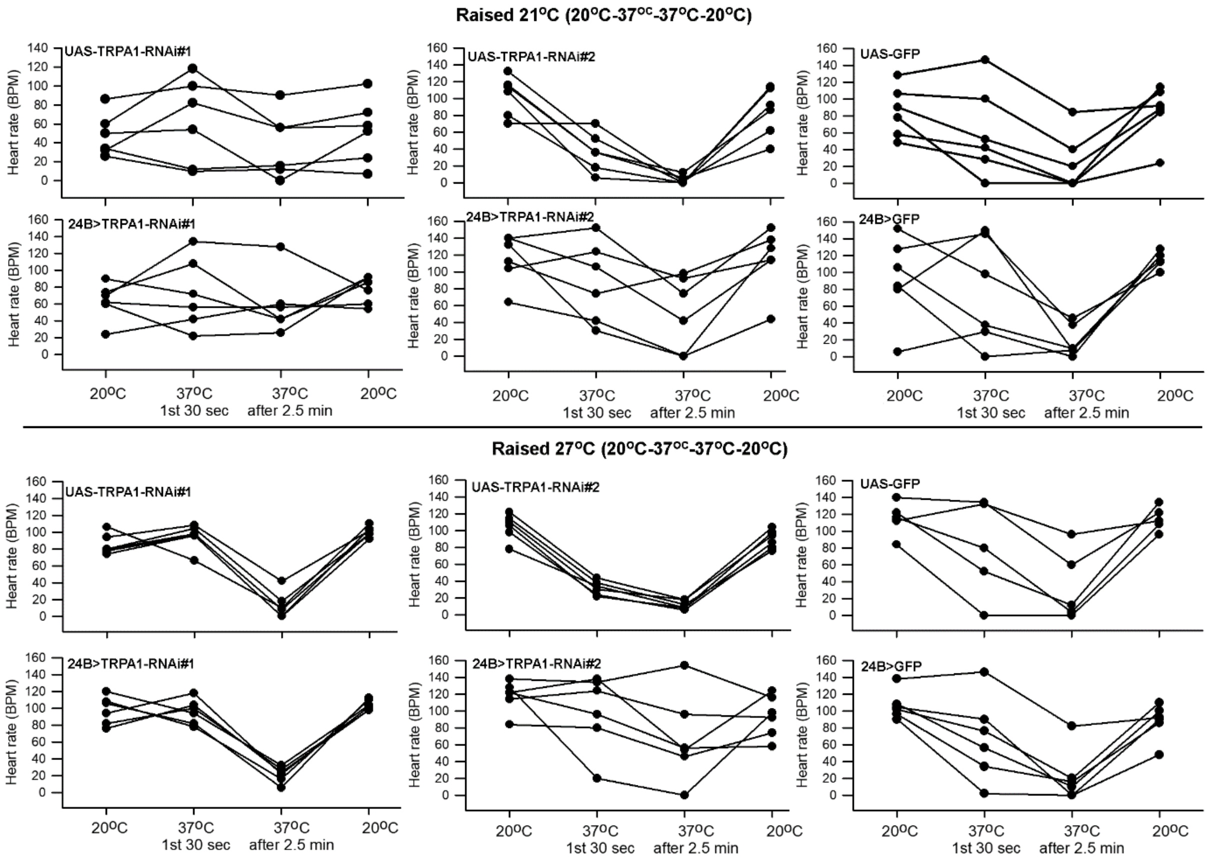

S4: *The heart rate for the dissected background and expression of TrpA1-RNAi in mesoderm for larvae raised at 21°C and 27°C for exposure to 37°C. The heart rate showed a lot of variation upon the initial exposure to 37°C for most of the strains; however, after two and half minutes the rates generally decreased for all the strains. The UAS-TrpA1-RNAi#2, 24>TrpA1-RNAi#2, UAS-GFP and 24B>GFP strains showed a lot of variation upon exposure 37°C for being raised at 21°C or 27°C. There is no significant difference from background strains compared to the ones crossed to the 24B strain.*

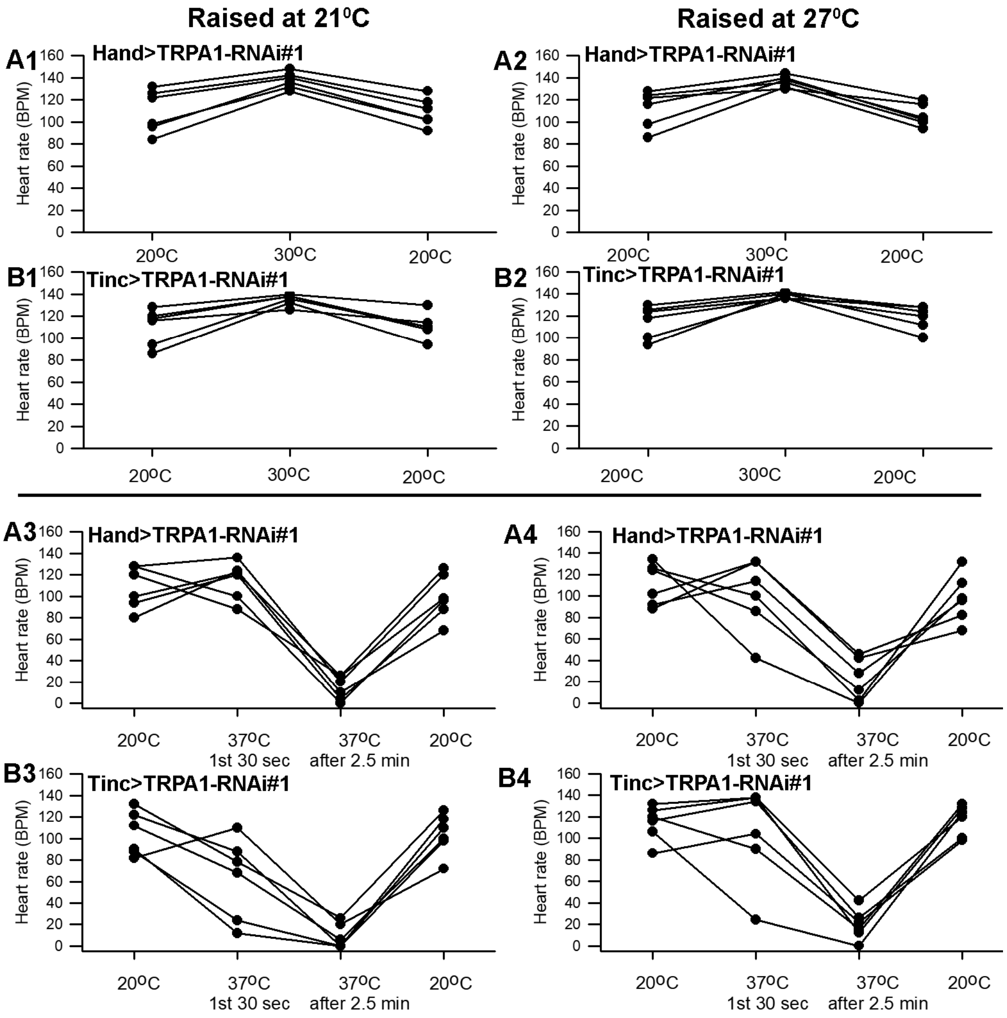

S5: *The heart rate for the dissected strains expressing TRPA-RNAi in heart specific tissue. The heart rate increased in* Hand4.2>TrpA1-RNA#1 (A1, A2) and Tinc>TrpA1-RNAi#1 (B1, B2) *from 21°C to at 30°C (P<0.05, Paired T-test) for both conditions of larvae raised at 21°C(A1, B1) as well as at 27°C (A2, B2). The stains* Hand4.2>TrpA1-RNAi#1 (A3, A4) and Tinc>TrpA1-RNAi#1 (B3, B4) *upon initial exposure at 37°C showed quite a bit of variation. However, at 37°C after 2 and half minutes all rapidly decreased heart rate. The same trends were present for the larvae raised at 21°C (A3, B3) as well as at 27°C (A4, B4). There is no significant difference from background strains compared to the ones crossed to the heart specific strains.*

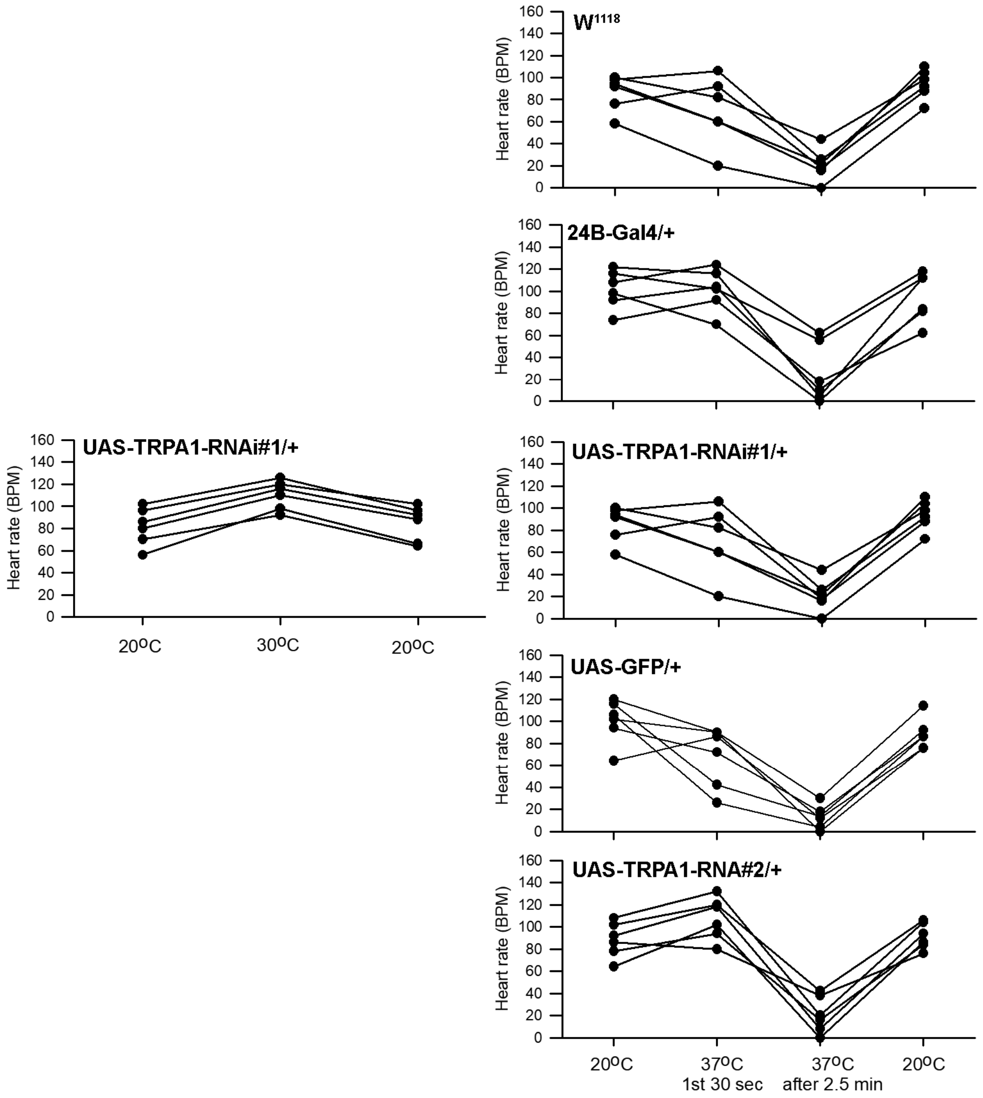

S6: Controls for the expression of the RNAi strains crossed with the W118 strain for dissected preparations. The UAS-TrpA1-RNA#1/+ showed similar trends as UAS-TrpA1 in an increase in heart rate at 30°C. The 24B>TrpA1-RNAi#1 as well as the backgrounds and controls (UAS-TrpA1-RNAi#1, UAS-TrpA1-RNAi#2, UAS-GFP, and 24B>GFP) all showed variation upon initial exposure to 37°C and showed a decrease after two and half minutes at 37°C. No significant difference occurred among the strains.
